# Impact of primary headache disorder on quality of life among school students in Kuwait

**DOI:** 10.1186/s10194-020-01124-3

**Published:** 2020-06-24

**Authors:** Jasem Y. Al-Hashel, R. Alroughani, S. Shauibi, A. AlAshqar, F. AlHamdan, H. AlThufairi, S. Owayed, Samar F. Ahmed

**Affiliations:** 1 Neurology Department, Ibn Sina Hospital, P.O. Box 25427, Safat, 13115 Kuwait City, Kuwait; 2 Department of Medicine, Faculty of Medicine, Health Sciences Centre, Kuwait University, P.O. Box 24923, Safat, 13110 Kuwait City, Kuwait; 3 Division of Neurology, Department of Medicine, Amiri Hospital, Arabian Gulf Street, 11013 Sharq, Kuwait; 4 Internal Medicine Department, Ministry of Health, Kuwait City, Kuwait; 5 Obstetrics and Gynecology Department, Ministry of Health, Kuwait City, Kuwait; 6 Neuropsychiatry department, Faculty of Medicine, Al-Minia University, P.O. Box 61519, Minia City, Minia 61111 Egypt

**Keywords:** Primary headache, Quality of life, Burden of headache

## Abstract

**Background:**

Primary headache disorders have being increasingly reported in younger populations. They can have significant effects on their quality of life and academic achievement and may cause significant distress to their families.

**Aims and objectives:**

To assess the burden of primary headache disorder and its impact on the quality of life on school student in Kuwait.

**Methods:**

**A** cross-sectional study was conducted among Kuwaiti primary and middle school students of both genders in randomly selected schools located in two governorates in 2018/2019 academic year. Headache-Attributed Restriction, Disability, Social Handicap and Impaired Participation (HARDSHIP) questionnaire for children and adolescents was used to assess the impact of primary headaches on the quality of life.

**Results:**

One thousand and ninety-one questionnaires were completed by primary and middle school students of both genders; of whom 466 students (girls 321 (68.88%) were diagnosed with primary headache disorders with mean age 11.98 ± 2.03 years. In the month prior to the survey, the effect of the headache was variable. The students lost a mean of 1.99 ± 2.015 days of school while they could not perform their usual activities for a mean of 2.84 ± 4.28 days. Their parents lost a mean of 2 ± 2.03 days of work because of headaches of their children and parents prohibited 5.7% of the students to engage in any activity due to their headaches. Difficulties in concentrations were reported as never sometimes (39.1%), often (24.8%), and always (26%). Majority of the students (51.5%) experienced a feeling of sadness ranging from sometimes to always. Most of the students (67.3%) struggled to cope with the headache and 22.4% were never able to cope. Additionally, 19.4% of students reported they did not want others noticing their headache.

**Conclusion:**

Primary headache disorder can have a significant impact on the quality of life in children. It can affect their engagement in activities and academic achievement. Implementing strategies to properly manage schoolchildren with primary headaches can have profound effects on their quality of life.

## Introduction

Among chronic pains experienced in childhood and adolescent population, headaches are staggeringly the most prevalent pain experienced in regard to lifetime prevalence [1]. Headaches can significantly lead to debilitated cognitive, emotional and recreational functioning in all areas of life ranging from their homes to their scholar activities [2]. It was found that school attendance was affected amongst children with headaches [3]. Additionally, the same children are more prone to develop psychiatric disorders such as depression and anxiety as well as other somatic symptoms such as abdominal pain [4–6]. These complaints are important to be documented as it was reported that pediatricians failed to recognize the emotional and behavioral impacts caused by their disease [7]. Moreover, a serious negative correlation was observed between the intensity and frequency of the pain with the quality of life [8]. Headache disorders are burdensome conditions has been reported in sprevious papers. The meaning of migraine is a burdensome condition is not univocal. There are at least six main themes that have been associated to the broad concept of burden and impact of primary headache as its prevalence, its overall impact (mostly defined as reduced QoL or disability), impact on work or school activities, impact on family life, interictal burden and disease costs [9].

Studies assessing the impact of headaches on the wellbeing and functioning of the inflicted are scarce worldwide especially among the pediatric and adolescent age groups; in particular, the Arab world including Kuwait have few studies of impact of headache. Thus, a research of this kind is conducted to shed light on the burden of such headache disorders. Once the burden is recognized, efforts can be implemented to improve not only the patients’ quality of life in the future leading to positive outcomes over time, but also their parents as it is also noted that their lives are limited with the responsibilities that are associated with taking care of their children [10].

## Methods

We conducted a cross-sectional study with a school-based sample whereby a questionnaire was distributed to primary and middle schoolchildren aged 7–16 years in governmental schools in Kuwait. In Kuwait the schools for girls are separated from school of boys. Equal numbers of boys and school girls are included in our study. Both of boys and girls schools were randomly selected from two major governorates in Kuwait; Al-Farwaniyah, the most densely populated and farther away from the center of the State of Kuwait, and Hawally, which is more urbanized and central in location. These two governorates were chosen to cover the geographic diversity of Kuwait.

The number of Kuwait students in academic year 2018/2019 according to the information from Kuwait Ministry of education in primary and middle schools is 127,653. Of those, 71,448 (55.97%) are girls and 56,205 (44.03%) are boys. We calculated the sample size to be 950 using a special formula. It was based on reported prevalence of headache from previous national and international epidemiological studies, which is around 54.4% and of migraine, 9.1% [11, 12]. Then, the sample was increased by 20% to overcome the problem of non-response and missing data.

Representative random sample of school classes was selected, stratified by grade (3rd, 5th, 7th, 9th), and school type (school for boys and schools for girls). So, the final selection of schools and school classes covered the age spectrum from childhood through adolescence and reflected Kuwaiti students of schools appropriately. Subjects were excluded if refused to participate, were non-Kuwaiti nationals, had history of medical or neurological disease other than primary headache or were absent on the day of the survey.

The survey used Lifting the Burden, Headache-Attributed Restriction, Disability, Social Handicap and Impaired Participation (HARDSHIP) questionnaire that was translated into Arabic [13]. The Child HARDSHIP for children aged 6–11 years and Adolescent HARDSHIP questionnaire for adolescents aged 12–17 years were used in this study. The HARDSHIP questionnaire has already demonstrated validity and acceptability in multiple languages and cultures including the Arabic Language. This questionnaire questions included sociodemographic, screening and diagnostic questions and enquiries of various domains and quality of life. The last part of the questionnaire included questions on the use of healthcare system in the past, medication use, and Headache-Attributed Lost Time Index questionnaire. Burden questions referred to the numbers of days missed from school, leaving school early or with impaired everyday activities due to headache, within the previous 4 weeks. Data were obtained from the children and adolescents themselves after explanation of the questions by physician of the study team. Questionnaire distribution and data collection were organized and conducted by physician supervisors during a school class as a paper-pencil version. To collect study data, well trained physicians conducted face to face interviews using Child and Adolescent HARDSHIP questionnaires. Written informed consent was obtained from all participants and their parents before the questionnaire was distributed. The participants were granted the right to decline participation at any time during data collection. Diagnoses were performed by HARDSHIP algorithm [14]. Confirmation of diagnosis was done by headache specialist applying ICHD-3 criteria [15].

The team leader reliably stored all completed questionnaires at the end of each day. Errors in data entry or diagnosis of primary headache were corrected by discussing them with the interviewer and a revisit was arranged if discrepancies could not be corrected. The team leader monitored and assisted researchers on a regular basis to resolve any problems and to review the completed questionnaires. The fieldwork was carried out over a period from 1/10/2018 till 1/1/2019.

Ministry of health and ministry of Education in Kuwait approved the study. Participants was given a simple explanation about the aim of the study being considered an ethical issue. All data were protected in accordance with the ethical guidelines of the Council for International Organizations of Medical Sciences and the principles in the Declaration of Helsinki [16, 17].

### Statistical analysis

The data from completed questionnaire were entered on IBM SPSS Statistics 20.0. Data entry was double-checked with inconsistencies reconciled by reference to the source documents. An error rate of 1.9% was identified. Proportions, 95% CIs, means, and standard deviations (SDs) were used to summarize the distribution of variables.

## Results

Of 1485 questionnaires that were distributed, 1091 students completed the questionnaire with a respondent rate of 73.4%. Females reported significantly more primary headaches as compared to males. Table 1 display the characters and burden of Primary Headache among school children with Primary Headache Disorders. Most of our cohort 77% used analgesic of their headache. With respect to severity, majority of students labeled their headaches as ‘quite bad’, whereas few of students experienced ‘very bad’ headaches. Headache reporters had to lose a mean of 0.99 days off school due to headaches and had to leave school early on a mean of 0.77 days.

**Table 1:** Characters and burden of primary headache among school children with primary headache disorders (No = 466)

Variables	Total school students with primary headache *n* = 466 m ± SD/No (%) [CI 95%]	Primary school students with primary headache *n* = 128 m ± SD/No (%) [CI 95%]	Middle school students with primary headache *n* = 338 m ± SD/No (%) [CI 95%]
**Gender:**			
• Male	145 (31.12%) [27.08–35.46]	23 (17.97%) [12.22–25.59]	216 (63.91%) [58.65–68.85]
• Female	321 (68.88%) [64.54–72.92]	105 (82.03%) [74.41–87.78]	122 (36.09%) [31.15–41.35]
**Mean Age in years**	11.98 ± 2.03	9.22 ± 0.99	13.03 ± 1.16
**Range**	7–17	7–12	11–17
**Number of headache days in the last four weeks**	6.24 ± 5.18	5.28 ± 4.82	6.58 ± 5.27
**Duration of attacks in hours in the last four weeks**	9.12 ± 2.51	8.82 ± 2.16	9.32 ± 3.11
**Number of analgesic days in the last four weeks**	3.26 ± 4.20	3.03 ± 3.45	3.35 ± 4.45
**Severity of headache**			
• Not bad	108 (23.18%) [19.57–27.22]	27 (21.09%) [14.87–29.00]	81 (23.96%) [19.71–28.80]
• Quite bad	277(59.44%) [54.92–63.81]	85 (86.94%) [84.68–88.91]	192 (65.80%) [51.48–61.98]
• Very bad	81 (17.38%) [14.20–21.10]	16 (12.50%) [7.74–19.44]	65 (19.23%) [15.37–23.78]
**Impact of primary headache over last 4 weeks before the survey**			
• Mean lost school days	1.99 ± 2.015	2.26 ± 0.20	1.92 ± 0.11
• Mean days of leave school early	0.77 ± 1.69	1.38 ± 0.12	1.79 ± 0.10
• Mean days could you not do things because of headache	2.84 ± 4.28	2.42 ± 0.22	4.78 ± 0.27
• Mean lost days of parents work because of student headache	2.00 ± 2.03	1.54 ± 1.00	2.15 ± 2.25

*m* mean, *SD* standard deviation, *CI 95%* confidence interval 95%

**Fig. 1:**
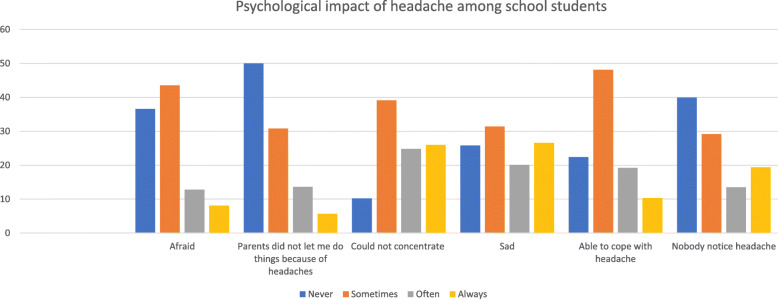
Psychological impact of headache among school students

The effect of the headache was variable in the preceding month (Fig. 1). Almost half of the students were sometimes afraid of having an attack (43.5%) while (8.1%) were always afraid. 5.7% of the sufferers were always prohibited by their parents to engage in any activity due to their headaches, while 50% never had any issue. Difficulties in concentrations were reported as never (10.2%), sometimes (39.1%), often (24.8%), and always (26%). Majority of the students (51.5%) experienced a feeling of sadness ranging from sometimes to always, on the other hand, 25% never experienced it. Most of the students (67.3%) struggled to cope with the headache as (22.4%) never was able to cope, while sometimes 48.1% could manage. The greater number of the students were comfortable with people noticing their headaches, in contrast to 19.4% who always did not want people to notice it.

**Fig. 2:**
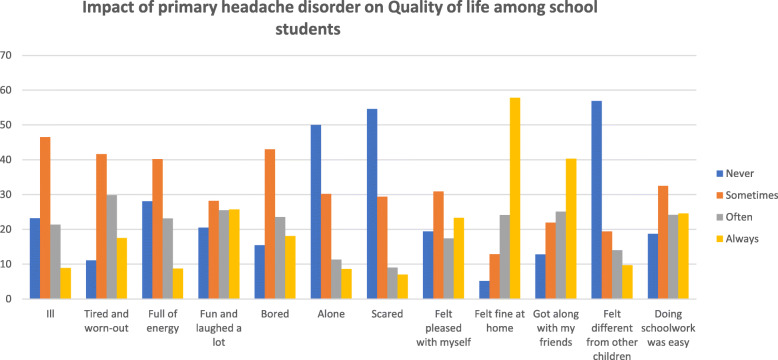
Impact of primary headache disorder on quality of life among school students

Primary headaches had a major impact on the student’s quality of life over the last month as shown in Fig. 2. Almost half of the students felt ill sometimes (46.3%), while the minority felt ill always (8.9%). While (11.1%) of the students never felt tired or torn out, (41.6%) felt tired sometimes, (29.8&) often, and (17.8%) always. On the other hand, almost a third of the students disclosed that they never felt full of energy (28.1%) while the remaining was majorly described as sometimes (40.2%). 20.5% of the sufferers struggled by never laughing or having fun, while the remaining were able to in almost equal numbers between sometimes, often, and always (28.2%, 25.5%, and 25.7%). Feeling bored was always experienced by 18.1% of the population, however, almost half of the remaining felt it sometimes (43%).50% of the children reported never feeling alone, followed by sometimes in (30.2%). Moreover, feeling scared resembled the previous numbers documented as never in (54.6%) and (29.4%) as sometimes. A striking (19,.4%) of the schoolers reported never feeling pleased with themselves while (23.3%) were always are. Always feeling fine at home was reported to reach as high as (52.9%) in contrast to (8.6%) ho never felt it. Less than half were always able to get along with their friends (40.3%), yet (12.8%) always struggled. Feeling different from the other children was witnessed always in (9.7%) of the students, nonetheless, (56.9%) never experienced it. When asked about having troubles in doing schoolwork, 18.7% of patients never found them to be easy, while 32.2% labelled them as sometimes easy.

## Discussion

A previously published population-based study in Kuwait estimated that one-year prevalence of primary headache disorders in children and adolescents was 19.4% [11]. Studies that estimate the impact of primary headache in children are scarce. Understanding the burden of headache disorders is important to assess health needs and to plan allocation of resources. Our current study may help to direct health-policy decision making and setting priorities. Assessment of the quality of life could have been done using health-related quality of life (HRQOL) measurement, however, it’s validity and applicability in headaches were not found in the literature [18].

Headache may result in significant disability, including missed school days, and extra-curricular activities, decrease participation in regular activities, and loss of productivity [19]. Approximately one-third of students in our cohort lose at least 6–7 school days, and consumed analgesics at least 7 days. Previous population-based studies reported that students missed on average 7.2 school days in the last 6 months due to migraine, which is comparable to our study where the mean was 0.99 days in the last month [20]. The burden of headache in children and adolescents in our results is in agreement with earlier results that revealed total scores of 17.8 to 44 days where pediatric headache patients were totally or partially disabled at home or at school because of their headache [21].

Majority of our cohort used symptomatic medications for headache on average 3.26 days a month, which is in agreement with earlier studies, which reported medication use among children in a variable percentage ranging from 30 to 80% [22, 23]. In a German study, 83.6% of the children used analgesics or anti-migraine drugs [24]. Preventive drug therapy for primary headache is not always needed in young headache patients to avoid the risk of side effects. Non-pharmacological treatments of headache disorders could be considered as a first line strategy in children and adolescents with primary headaches [24].

In addition, a total of 34.7% of our study participants reported not getting along with their friends ranging from never to sometimes getting along. This form of social anxiety was also paralleled in another research in Italy [25]. Moreover, the extent of headaches can lead to lack of attention and concentration which was reported in our study participants as well as those in other researches [6].

Headaches also affect mental health. It was observed that about 42.7% of the children reported being often or always sad in our study. A similar number was translated in terms of their energy, concentration, enjoyment, self-worth and functional levels. It was similarly noted that headaches carry the burden of multiple comorbidities, in particular, psychological ones such as depression and anxiety amongst children of Europe and Korea [25–31].

### Strengths and limitations and of the study

Our study represents aimed to exhibit the relationship between pediatric headache and the quality of daily life. To our knowledge similar studies have never been carried out in the Middle East. We demonstrated variable psychological burdens that will help us raise the awareness of pediatricians, teachers, and family member in order to detect and intervene earlier. Our study sample was collected from the biggest governorate in Kuwait in order to eliminate location as a confounder. The results of our study may be limited by recall bias, which is usual in most of the studies using the questionnaire.

## Conclusions

Recognizing the psychosocial effects induced by headaches is vital for the development of the best clinical and holistic care for the patients. For that reason, we believe assessing the child’s subjective experience with the headaches in regard to the quality of life is crucial. In addition, it serves as a ground basis for future research investigations in attempt to implement the best interventions to tackle all the emotional and behavioral issues.

## Data Availability

Data are available on request from the Department of Neurology, Ibn Sina Hospital, Safat, Kuwait. P.O.Box 25427, 13115 Safat, Kuwait City, Kuwait.

## Abbreviations

HARDSHIP: Headache-Attributed Restriction, Disability, Social Handicap and Impaired Participation
ICHD-III: International Classification of Headache Disorders III
