# Correction to: Impact of primary headache disorder on quality of life among school students in Kuwait

**DOI:** 10.1186/s10194-021-01217-7

**Published:** 2021-02-14

**Authors:** Jasem Y. Al-Hashel, R. Alroughani, S. Shuaibi, A. AlAshqar, F. AlHamdan, H. AlThufairi, S. Owayed, Samar F. Ahmed

**Affiliations:** 1 Neurology Department, Ibn Sina Hospital, P.O. Box 25427, Safat, 13115 Kuwait City, Kuwait; 2 Department of Medicine, Faculty of Medicine, Health, Sciences Centre, Kuwait University, P.O. Box 24923, Safat, 13110 Kuwait City, Kuwait; 3 Department of Medicine, Division of Neurology, Amiri Hospital, Arabian Gulf Street, 11013 Sharq, Kuwait; 4 Internal Medicine Department, Ministry of Health, Kuwait City, Kuwait; 5 Department, Obstetrics and Gynecology, Ministry of Health, Kuwait City, Kuwait; 6 Department, Neuropsychiatry, Faculty of Medicine, Al-Minia University, P.O. Box 61519, Minia City, Minia 61111 Egypt

**Correction to: J Headache Pain 21, 80 (2020)**

**https://doi.org/10.1186/s10194-020-01124-3**

Following publication of the original article [1], we were notified of a typo in the third author’s name.

Originally published name: S. Shauibi

Corrected name: S. Shuaibi

The original article has been corrected.
